# How Can Cities Respond to Flood Disaster Risks under Multi-Scenario Simulation? A Case Study of Xiamen, China

**DOI:** 10.3390/ijerph16040618

**Published:** 2019-02-20

**Authors:** Yijun Shi, Guofang Zhai, Shutian Zhou, Yuwen Lu, Wei Chen, Jinyang Deng

**Affiliations:** 1 School of Landscape Architecture, Zhejiang A&F University, Hangzhou 311300, China; 2 School of Architecture and Urban Planning, Nanjing University, Nanjing 210093, China; shutian_zhou@smail.nju.edu.cn (S.Z.); yuwen_lu@smail.nju.edu.cn (Y.L.); 3 School of Geography and Ocean Sciences, Nanjing University, Nanjing 210093, China; chenw.nju@gmail.com; 4 School of Natural Resources, West Virginia University, Morgantown, WV 26506, USA; jinyang.deng@mail.wvu.edu

**Keywords:** flood disaster, risk assessment, multi-scenario simulation, response strategies, Xiamen city

## Abstract

Flood disasters often have serious impacts on cities. Disaster prevention and mitigation schemes for flood disasters must be based on risk assessment. We constructed an indicator system for flood disaster risk assessment from the aspects of hazard factors, sensitivity to the environment, disaster vulnerability, flood disaster prevention, and resilience. Then we add the precipitation factor as a scenario parameter to the assessment of flood disasters, in order to assess the flood disaster risk under annual average precipitation scenarios, multi-year flood season average precipitation scenarios, and large typhoon precipitation scenarios. Xiamen is one of the cities with more serious flood disasters. We select Xiamen as an example and refer to existing indicators of flood disaster assessment. The results show that: (1) the coefficient of variation of flood disasters in Xiamen under the impact of large-scale typhoon precipitation is large; (2) the drainage and flood control capacity of Xiamen is generally insufficient, and the risk in the old city is high; (3) there are many flood-prone locations in Xiamen. Underpass interchanges, underground spaces, and urban villages have become the new key areas for flood control; and (4) the flood risk in the northern mountainous areas of Xiamen is the highest. Based on the assessment results, we further delineate the urban flood control zones and propose corresponding countermeasures. The study expands the research on flood disaster risk assessment, and also provides reference for relevant cities to deal with flood disasters.

## 1. Introduction

As the main body of human social activities, cities have always suffered from various types of disasters, especially natural disasters have caused very large losses to human society. According to the International Emergency Disaster Database (EMDAT) [1], from 1900 to 2017, the world has suffered 12,547 major natural disasters with a total of 22,989,400 deaths and economic losses of up to $290.28 billion. Among these disasters, the flood disaster occurred the most frequently, resulting in the highest number of deaths and economic losses. It can be said that flood disaster is one of the most serious natural disasters affecting human society [2]. For example, in 2012, Hurricane Sandy caused widespread flooding of road networks along the coast of New York City, where firefighting vehicles could not reach fire sites and 122 houses were burned. In 2017, Hurricane Irma hit Florida, resulting in 123 deaths in the state, including 14 from a local nursing home. The hurricane has also caused large-scale rescue interruptions and power outages [1]. On 13 June 2018, Shenzhen, China, recorded its historically greatest rainfall, which caused serious floods with tremendous economic losses. Six people were killed, and more than 20,000 people were forced to evacuate [3].

As one type of natural disasters, urban flood disasters are often caused by a short-term heavy rainfall or a long-term precipitation that accumulates a massive amount of water that cannot be easily drained in time. Flood disaster often cause serious disruptions to cities, including loss of life, damage to property, and destruction of public infrastructures, which usually cause serious environmental pollution and more negative social impacts. Urban flood disaster is a complex problem, so the flood control and disaster mitigation plan must be based on the flood disaster risk assessment [4]. Flood disaster risk assessment is one of the most effective ways to reduce flood occurrence and disaster losses [5], and it is also the key technical support for urban disaster prevention and mitigation assessment decision [6], and thus has become one of the hotspots that draws increasing attention from researchers. Understanding the features of flood disasters helps us to understand the causes of flood disasters. Therefore, finding an appropriate method to assess flood risk is the premise of flood risk management. This paper aims to establish an applicable model to assess urban flood disaster risk for achieving an optimal outcome. A scientific understanding of urban flood disasters will allow us to plan in advance of the urban planning and construction, and minimize the damage caused by the disaster.

Our paper offers three main contributions. First, we factor precipitations a scenario parameter into the assessment of flood risk to obtain the results of flood disaster risk under multi-scenario simulations. Secondly, we combine the historical flood disaster data with the flood disaster risks under different scenarios to analyze the location points of flood disasters. Finally, based on the simulation results, we delineate the urban flood control zones and propose corresponding countermeasures.

The rest of the paper is organized as follows. In Section 2, we review the current indicators and methods for assessing flood disaster risk. In Section 3, we introduce the study area and methods used in this study. In Section 4, we use Xiamen as an example and assess the flood disaster risk under different scenarios. On this basis, we combine with the historical floods locations in Xiamen, and obtain the locations that are prone to flood disaster under different scenarios. In Section 5, based on main research findings, we propose the flood control zones as well as relevant planning response strategies of Xiamen. In Section 6, we draw the main conclusions.

## 2. Literature Review

The flood disaster assessment mainly consists of two aspects: flood disaster risk assessment (FDRA) and flood disaster damage assessment (FDDA) [7]. The FDRA mainly focuses on the quantitative analysis of the causes, probability and intensity of flood disaster [8,9,10,11]. Different scales of precipitation may generate different flood risks. Through FDRA, we can clearly understand the level and spatial distribution of flood disaster risk, so that we can effectively manage and control the flood risks. The FDDA is to quantitatively analyze the loss of life, damage to property, and impacts on social economy or the environment caused by flood disaster [12,13,14]. In recent years, scholars have gradually realized that flood disaster should be analyzed from the perspective of risk, and flood disasters risk research is gradually developing from traditional disaster prediction to disaster risk analysis. An examination of the existing research progress of FDRA shows that the FDRA methods have evolved from the macro level to the micro level, from a focus on the cause mechanism of the disaster to the application of the disaster statistics, and from the construction of the floods indicator system to multi-scenario simulations [15]. FDRA methods, according to the development stages of methods, can be summarized as mathematical statistics method [16], index system method [17,18], simulation analysis method [19,20,21] and spatial analysis method [22,23].

The mathematical statistics method is mainly utilized on the basis of historical disasters data in the study area. Through statistical analyses involving the frequency of disaster occurrences, and the area and population affected, the pattern of disasters occurrence can be found, and the relationship between the probability of disasters occurrence and associated factors can be established, so that the disasters risk can be evaluated in advance. Therefore, this method is also called historical disaster probability statistics method. Benito et al. [24] used historical floods data and a combination of geology, history, statistics and other multidisciplinary approaches to assess flood risk. Nott [25] proposed the long-term sequence of floods data as the basis for flood risk assessment in a regional scale. Qin and Jiang [26] used historical flood data to analyze the risks of flood disaster in the lower reaches of the Yangtze River in China. Huang et al. [27] conducted disaster damage assessment based on long-term disaster data. Liu and Shi [28] proposed a regional flood risk assessment method based on the historical data of the Yangtze River Basin. Mathematical statistics method is a simplified method, which is mainly based on historical disaster probability statistics in the process of calculation, and it does not require background data (e.g., such as topography, landform, and water system), and can be calculated based on statistics of historical data. However, due to the lack of availability and quantifiability of statistical data, this method often encounters fewer samples and incomplete sample content in the calculation process. This may lead to results being less reliable or robust. Moreover, the results calculated by this method may also be divergent from the actual situation, or even be inconsistent with the reality [29].

The index system method is a static analysis method. The principle of this method is to select certain indicators based on the characteristics of disasters, and then process the raw data through mathematical methods to obtain the probability of disaster risks for the study area. This method is also known as the comprehensive evaluation method [30]. For flood disasters, researchers usually select precipitation, river network, elevation, population, economy, and infrastructure as indicators. Through mathematical evaluation methods, the risk of flood disaster in an area is assessed from the perspectives of the risk of the hazard factors, the sensitivity of the environment, the vulnerability and vulnerability of the hazard [31,32]. The mathematical methods used most commonly in the calculation process of indicator system methods mainly include the fuzzy comprehensive evaluation method, the grey relational analysis method, the analytic hierarchy process and the principal component analysis method. The main difference between these methods lies in the use of different criteria for determining the weights of selected indicator. Common weight determination methods include the principal component analysis method, the analytic hierarchy process method and the entropy weight method. The analytic hierarchy process method is subjective, and its calculation results are easily influenced by the randomness in the evaluation process and the subjective uncertainty and cognitive ambiguity of the evaluation experts. The entropy method and the principal component method are relatively objective. However, the principal component method may result in the loss of more information lost during the calculation process. Since the indicator system method can reflect the flood disaster risk on a macro scale, it has been widely used in disaster risk analysis and assessment [32].

The scenario analysis method is to propose various key hypotheses based on the current situation of disasters, and then to construct or modify relevant mathematical models by constructing algorithm models and developing related software models to simulate urban flooding processes [33]. Based on different parameter values, we can construct different flood disaster scenarios for specific evaluation and analysis. With the development of computer technology, the scenario analysis method based on algorithm and software analysis has become one of the main method to study the risk of urban flood disaster. Thus far, a variety of urban flood disaster simulation models have been formed. Of these models, the storm water management model (SWMM) developed by the U.S. Environmental Protection Agency, the MIKE series model developed by the Danish DHI Hydraulic Research Institute and the InfoWorks software developed by the National Institute of Wallingford in the UK are widely used [34]. The construction or revision of hydrological and hydrodynamic models is the key to the simulation of urban flood disaster. Some researchers simulate urban flooding processes by constructing new models or improving existing models [35,36]. By establishing relevant mathematical models, it is the mainstream research method to simulate the flood disaster and the disaster losses [37,38,39]. The flood risk assessment based on multi-scenario analyses can better reveal the spatial and temporal changes in flood evolution, and to some extent reflect the physical processes and mechanisms of flood disasters. However, this method lacks system reliability and pays less attention to the analysis of risk levels.

The spatial analysis method uses the digital terrain technology and spatial analysis function of geographic information system (GIS) software to determine the risk of the water accumulation area and flood disaster. The method takes the principle of water flowing from high pressure to low pressure as the basis for calculation. Since floods have gravitational characteristics of flowing from high to low, so spatial simulations on flooding often rely on the digital elevation model (DEM). Abdalla et al. [40] used GIS, DEM and fuzzy evaluation methods to assess the flood disaster risk and classify flood risk levels in the Red River region of Southern Canada. Santha [41] established a flood disaster assessment model that is suitable for the coastal areas of India. Liu et al. [42] constructed a flood disaster assessment model of the Bowen Basin in Australia using GIS, which directly reflect the floods situation and can be used for real-time monitoring of flood disaster. However, due to its simulation range being too wide, it is difficult to obtain high-precision risk assessment results.

From the existing research on the flood risk assessment, the index system method is still the most commonly used method so far, and it is also a relatively comprehensive method to systematically assess the risk of flood disasters [43]. At the same time, in order to overcome the shortcomings of a single method, a series of comprehensive methods such as multi-model collection and multi-model comparison analysis emerged as one of the most effective approaches for flood disaster risk analysis. For example, in order to solve the spatial and temporal heterogeneity of flood disaster risk, a spatial comprehensive analysis method based on the combination of flood evolution hydrodynamic model, remote sensing (RS) and GIS software are used [44]. Taking Xiamen as an example and referring to the existing indicators for flood disaster assessment, we construct an index system for the risk assessment of flood disaster in Xiamen. In the meanwhile, we propose to add multiple scenario factors as parameter factors to the assessment of flood disaster in Xiamen under different scenarios. Based on the results of the assessment, we further propose the division of flood control zones and the corresponding coping strategies. In addition, considering that the measurement units of various indicators of flood disaster risk assessment are not uniform, in order to solve the homogenization problem of different quality indicators relatively objectively, we use the entropy method to determine the weight of different evaluation indicators. On the one hand, this research verifies the scientificity and feasibility of the methods constructed in this paper. On the other hand, it also serves an example for the flood risk assessment for other similar cities.

## 3. Materials and Methods

### 3.1. Overview of the Study Area

**Figure 1:**
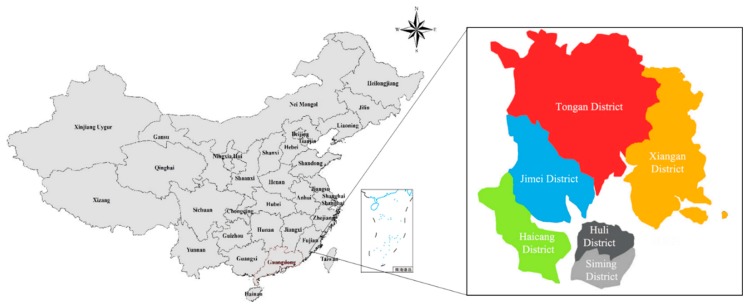
The administrative division map of Xiamen

Xiamen City is located in the southeast coast of Fujian Province in China (see Figure 1). It is the main central city and port city in the southeastern coastal areas of China. In 2017, the resident population of Xiamen was 4.01 million, the urbanization rate reached 89.1% and its GDP was 435.18 billion CNY (approximately $64.95 billion (using the rate 1 to 6.7, estimated)) [45]. Affected by hazard factors, such as climate, topography, and water system, flood disasters have always been one of the major disasters affecting Xiamen. Xiamen features a typical coastal hilly landform. Topographically, Xiamen is dominated by hills, especially in the central part of it. The terrain outside the island descends from the northwest to the southeast and forms a ladder-like landscape of hills and mountains, terraces, and plains. Xiamen has a subtropical maritime monsoon climate with an average annual rainfall of 1388 mm. Affected by topography, the annual precipitation shows an overall trend that decreases from southeast to northwest with a high variability across seasons and locations.

Typhoons are the main source of flooding to the city. Since 1949, large-scale typhoons have caused the five most serious floods in Xiamen’s history with serious casualties and economic losses. Flood disasters in Xiamen mainly occurred in the flood season (May to October of each year), which peaked in September. In recent years, with the continuous construction and improvement of urban water conservancy facilities and increasing enforcement of flood control standards, Xiamen’s ability to resist floods has been largely strengthened. However, the city is still threatened by extreme weather.

### 3.2. Methods

The FDRA refers to the analysis of the possibility and consequences of flood disasters based on the risk theory, which provides a basis for the development of urban flood control and related planning activities. This paper mainly draws on the theory of natural disaster risk in constructing the method of flood disaster analysis and assessment, and evaluate the disaster risk from four aspects: risk, sensitivity, vulnerability, and disaster prevention and mitigation ability [43]. At the same time, given the precipitation as the main trigger of the flood disaster, we use this factor as the scenario parameter to estimate the flood disaster risk under different precipitation scenarios. Considering the difference of the precipitation conditions, we constructed three precipitation scenarios: annual average precipitation, mean precipitation during flood season, and precipitation during the typhoon season. Based on these three precipitation scenarios, we have further obtained three assessment results of flood disaster risk with GIS software. The specific research methods are as follows:

#### 3.2.1. Index System

Generally speaking, FDRA mainly involves four aspects: hazard of disaster factors, sensitivity to environment, vulnerability of the disaster-bearing body, and the city’s disaster prevention capability [46,47]. Specifically, the hazard of disaster factors mainly reflects the intensity and frequency of disaster occurrences. Considering the causes of flood disasters, in this paper, we mainly choose several main factors, including precipitation, reservoir distribution, and capacity. Environmental sensitivity refers to the difficulty and probability of environmental problems in the ecological environment when encountering interference. Sensitivity to the environment of the flood disaster is relates to the geographical environment, geological conditions, and climatic conditions, etc. (e.g., in this paper, the elevation, slope, cultivated area, and river network density are selected). The vulnerability of the disaster-bearing body refers to the entities affected, where in the population size, economic development level and the building quality of the city can reflect the vulnerability of the city. Keeping the disaster prevention ability and disaster level constant, a city with a higher population density would be more likely to suffer more casualties. Likewise, a city with a high level of economic development would be more likely to be seriously affected economically. In this paper, when assessing the vulnerability of the disaster-bearing body for flood disasters, we also selected three indicators: population vulnerability, economic vulnerability and building vulnerability. Finally, the city’s disaster prevention capability is judged based on the construction and distribution of evacuation sites, urban rescue capabilities, and emergency management capabilities. For flood disasters, the city’s disaster prevention capabilities are mainly reflected in rainwater pipe network density, road network density and urbanization level [47]. In this paper, two indicators—urbanization level and road network density—are selected to assess the urban comprehensive response capacity. On the one hand, these two indicators directly determine the strength of the city’s ability to respond to disasters. On the other hand, the level of urbanization also indirectly or directly affects the government’s investment in infrastructure such as flood control and flood prevention, and further affects the overall strength of the city’s disaster prevention and mitigation capabilities.

When constructing the index system of FDRA in Xiamen, we also take into account these four aspects and use the entropy weight method to assign weights to all factors. The specific index system and weight values are as follows (see Table 1).

It should be noted that the units and attributes of selected indicators are different, which need to be standardized for calculation. There are two types of attributes in the index system: positive index vs. negative index. If the index value is larger, the measured risk level value is larger, then such indicators are referred to as the positive index. Otherwise, they are called the negative index.

The normalized formula for the positive index:

(1) $$A_{i}=\frac{a_{i}-\left\{a_{min}\right\}}{\left\{a_{max}\right\}-\left\{a_{min}\right\}}\times 100,$$

The normalized formula for the negative index:

(2) $$A_{i}=\frac{\left\{a_{max}\right\}-a_{i}}{\left\{a_{max}\right\}-\left\{a_{min}\right\}}\times 100,$$

$\left\{a_{max}\right\}$ and $\left\{a_{min}\right\}$ are the minimum and maximum values of each evaluation index in all years.

#### 3.2.2. Calculation of Indicator Weights

In order to reduce the subjective influence on the weight determination, we use the entropy weight method to determine the weights of the evaluation indices. The entropy weight method has the feature of strong objectivity. Compared with methods, the weights obtained by the entropy weight method have higher precision and more objectivity, and can better reflect the influence of the evaluation indicators on the results. The models are as follows [47]:

The original data form the matrix *X*:
  (3) $$X=\left[\begin{array}{ccc} X_{11} & \cdots & X_{Im}\\ \vdots & \ldots & \vdots \\ X_{n1} & \cdots & X_{nm} \end{array}\right],$$
By normalizing the raw data, a new matrix *Y* is obtained:
  (4) $$Y=\left[\begin{array}{ccc} Y_{11} & \cdots & Y_{Im}\\ \vdots & \ldots & \vdots \\ Y_{n1} & \cdots & Y_{nm} \end{array}\right],$$
Then the entropy value is derived as:
  (5) $$e_{j}=-k\sum_{t}\sum_{i}P_{ij}\ln P_{ij},$$
  (6) k=1/ln(t×n),
  (7) $$P_{ij}=Y_{ij}/\sum Y_{ij},$$
The weight *Wj* is calculated based on the entropy value:
  (8) $$W_{j}=\frac{1-e_{j}}{\sum_{j=1}^{m}\left(1-e_{j}\right)},$$
  where *X_ij_* is an element in matrix *X*, *t* is the number of years, *m* is the number of indicators, *n* is the number of samples and *e_j_* is the entropy value.

#### 3.2.3. Methods of Multi-Scenario Flood Risk Assessment

Based on the flood risk assessment indicators and calculated weights, the formula for the assessment of flood disaster risk is defined as follows:

(9) $$R=\sum_{i=1}^{n}F_{i}W_{i},$$

Taking into account that some of the indicators in the existing evaluation system (e.g., precipitation factors) are used as the average, the results of the assessment reflect more of the disaster risk in most states. Therefore, in this paper, we take the multi-scenario factors as parameter factors into the assessment of flood disaster risk to assess the risk of urban flood disasters under different scenarios. Then we make a correction to Equation (9):

(10) $$R=\sum_{i=1}^{n}\beta_{\theta }F_{i}W_{i},$$

(11) $$\beta_{\theta }=\left\{\beta_{\theta }|\theta =1,2,3,\ldots ,m\right\},m\in N*$$

where *β* denotes the scenario parameters. In this paper, we consider that the flood disaster is mainly affected by precipitation. Therefore, the precipitation factor is used as the scenario parameter to estimate the flood disaster risk under three precipitation conditions. *F* represents each indicator factor. *W* represents the weight of each indicator. *R* represents the risk of flood disaster.

Based on the comprehensive assessment results, we can grade the risk levels (Equation (12)) and plan for different flood disaster risk zones. In our article, we mainly use the Natural Breaks method to classify disaster risk levels. The Natural Breaks method is based on the natural grouping inherent in the data. The classification interval is identified, the similarity values can be optimally grouped, and the differences between the classes can be maximized:

(12) $$V=\left\{V_{1},V_{2},V_{3},\cdots ,V_{N}\right\},N\in N*,$$

## 4. Results

### 4.1. Hazard Analysis of Disaster Factors

**Figure 2:**
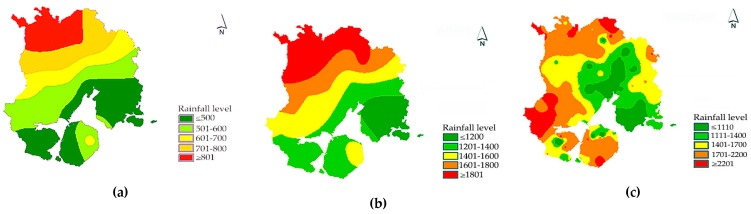
Simulation of precipitation in different scenarios in Xiamen: (**a**) precipitation simulation under multi-year average precipitation scenarios; (**b**) precipitation simulation under the flood season scenario; and (**c**) precipitation simulation under the typhoon transit scenario.

The flooding disaster factors are mainly considered from the perspectives of precipitation factors and flood factors. In the case of precipitation factors, we use the precipitation factor as the scenario parameter, and select three different precipitation scenarios (i.e., average annual precipitation, average precipitation during flood season and precipitation during typhoon) to simulate the flood disaster risk of Xiamen. (1) The average annual precipitation of Xiamen from 2005 to 2015 is selected to represent the situation under normal precipitation conditions. (2) The average annual precipitation during flood season (May to October of each year) in Xiamen during the same period is used to represent the general disaster situation. (3) The precipitation during the period of large typhoon (i.e., the Meranti typhoon in this paper) is selected to represent a scenario under extreme disaster conditions. Through the analysis of the precipitation data from the Xiamen Meteorological Bulletin from 2005 to 2015, we use GIS software to simulate different precipitation scenarios. It can be seen from the simulation results (see Figure 2) that the average annual precipitation and the average precipitation during the flood season show a trend of decreasing from the northwest to the southeast, and the precipitation under the typhoon scene is obviously affected by the typhoon path. From the spatial distribution of precipitation, the rainfall level under the typhoon scenario is the highest, and the precipitation in most areas exceeds the highest precipitation in scenario 1 and scenario 2.

**Figure 3:**
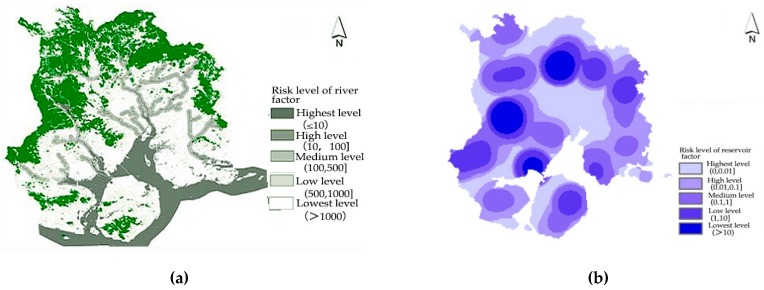
Analysis of flood factors in Xiamen: (**a**) analysis of river buffers; and (**b**) analysis of reservoir buffers.

In terms of flood factors, two indicators are considered: reservoir storage capacity and distance to the river. In general, the probability of a flood disaster in a certain area depends largely on the distribution of river networks in the region. The closer the distance to the rivers and lakes, the higher the risk of flood disaster would be. At the same time, the more the river water flow is, the larger the reservoir capacity is, thus the greater the impact range in the event of a flood. In this paper, the buffer zones of different river networks and reservoirs are used to demonstrate the impact of river networks on flood disaster. Different buffer widths represent the difficulty risk level affected by floods in different sections. From the analysis results (see Figure 3), the risk of river network in the dense areas is significantly higher, and the risk in the coastal areas is higher than that in the inland areas.

### 4.2. Sensitivity Analysis of the Environment

**Figure 4:**
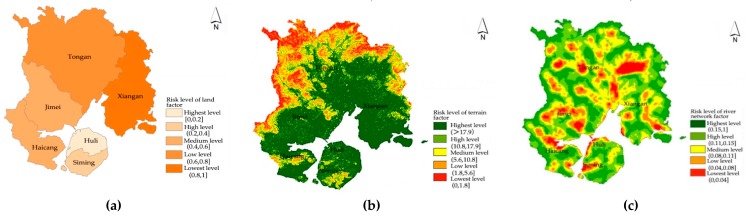
Sensitivity analysis of the environment in Xiamen: (**a**) analysis of land factor; (**b**) analysis of topographic factor; and (**c**) analysis of river network factor.

The sensitivity to environment of the flood disaster is examined from three perspectives: land factor, topographic factor and river network factor. (1) Land factor: Different types of land use affect the natural water regulation capacity and the runoff of rainwater on the surface differently. The soil in the forest area has strong water seepage ability, and it is not easy to form stagnant water. The water seepage capacity of agricultural and forestry land is moderate. The ground surface of the urban built-up area is dominated by a hard surface and the water seepage capacity is the worst. However, on the city scale, it is relatively difficult to obtain the data on the impervious surface of the road. This paper uses the proportion of cultivated land is used to indicate the water seepage capacity of the land factor and obtain the evaluation results of the land factors. From the assessment results (see Figure 4), we can find that the land in the downtown area of Xiamen has the worst water seepage capacity. As a result, the risk is higher than that in several areas outside the central city. (2) Terrain factor: The terrain of Xiamen is dominated by plains, terraces, and hills, and the terrain slopes from the northwest to the southeast. Mountainous terrain affects local precipitation, and in steep terrain, it is more susceptible to risks such as mudslides during torrential rains. Based on the DEM data of Xiamen, the two factors of elevation and slope are superimposed and analyzed, and the analysis results of topographic factors are obtained. It can be seen from the evaluation results (see Figure 4) that the risk of topographic factors in Xiamen gradually decreases from the northwest to the southeast. (3) River network factor: The water system in Xiamen is relatively complicated. The slope of the tributaries of the northern part of the mountain is large, the water is flooded quickly, the terrain in the southern part of the city is flat, and the coastal terrain is low. In view of this, we carry out surface runoff simulation and water flow length calculation for Xiamen. On this basis, river network extraction and river network density analysis is carried out, and the Xiamen river network density map was finally obtained (see Figure 4).

### 4.3. Vulnerability ANALYSIS of the Disaster-Bearing Body

**Figure 5:**
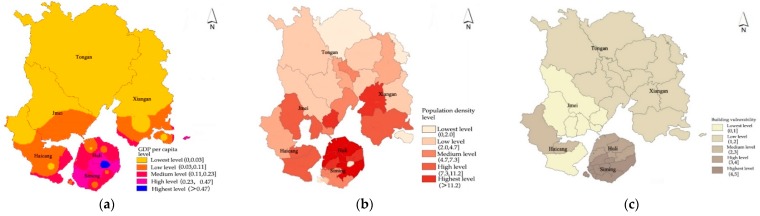
Vulnerability analysis of disaster-bearing bodies in Xiamen: (**a**) analysis of economic vulnerability; and (**b**) analysis of population vulnerability; (**c**) analysis of building vulnerability.

The vulnerability of the disaster-bearing body mainly refers to the loss level of people’s lives and property when the city is threatened by a flood disaster. It is related to the population of the area, the concentration of property, and the performance of the building. Holding other conditions constant, an area with a greater population density, higher GDP per capita, and worse building quality is more likely to suffer serious damages from flooding. Therefore, in this paper, we consider the vulnerability of the flood disaster risk from the perspectives of population vulnerability, economic vulnerability and building vulnerability. Specifically, four indicators are identified: population density, GDP per capita, building quality and building age. As Figure 5 indicates, Xiamen’s GDP per capita, population density and building quality all exhibit a consistent pattern that increases from the north to the south. The central part of the city with the highest GDP, highest population density, and best building quality is most vulnerable to flooding.

### 4.4. Analysis of the Disaster Prevention and Resilience

**Figure 6:**
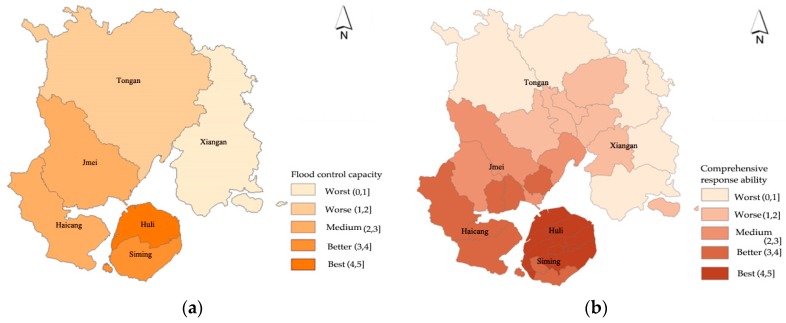
Analysis of the disaster prevention of Xiamen: (**a**) analysis of the flood control capacity; and (**b**) analysis of the comprehensive response ability.

Under the same disaster conditions, the flood control capacity determines the magnitude of the city’s losses in a flood disaster. The higher the city’s flood control capacity is, the smaller the damage caused by flooding. The disaster prevention capability of flood disasters is mainly determined by two aspects: the flood control capacity and the comprehensive response capacity. The former refers to the construction of disaster prevention infrastructures and drainage engineering measures, while the latter refers to the city’s ability to respond to disaster risks, which is related to the urban socio-economic level and the level of municipal facilities. Due to the impact of infrastructure and economic level (From the economic level and infrastructure status of Xiamen City, the Siming District, and Huli District are the core urban areas of Xiamen. The economic level is the highest, and the economic aggregate accounts for more than 50% of Xiamen (from 2005 to 2015). Meanwhile, the infrastructure of these two regions is relatively complete, and the investment in infrastructure assets accounts for more than 30% of the city’s total investment in assets (from 2005 to 2015). The economic aggregates of Jimei District, Haishu District, Xiang’an District, and Tong’an District are relatively low, and the infrastructure level is relatively low compared with the first two districts), the central urban area of Xiamen has the strongest flood control and drainage capacity and comprehensive response capacity, while the disaster prevention ability of Xiangan District and Tongan District in the urban fringe area is relatively weaker (see Figure 6).

### 4.5. Assessment and Analysis of Flood Disasters under Different Scenarios

As aforementioned, this paper constructs three flood risk assessment scenarios using the precipitation as a scenario parameter, and analyzes the flood risk levels for Xiamen under the three different precipitation scenarios. Each scenario is described below:

• Flood risk assessment under the multi-year average annual precipitation scenario.

**Figure 7:**
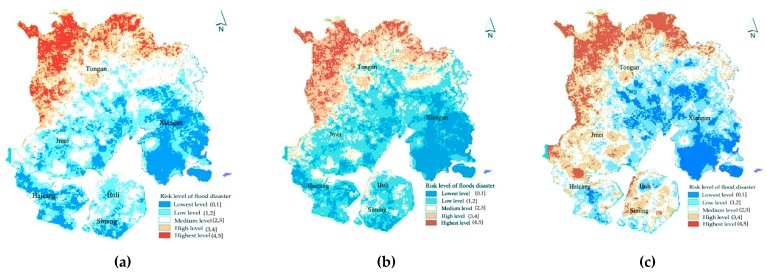
Risk assessment of flood disasters under different scenarios in Xiamen: (**a**) assessment results under the average annual precipitation scenario; (**b**) assessment results under the average precipitation during the flood season scenario; and (**c**) assessment results under the precipitation during typhoon scenario.

The average annual precipitation data of each site recorded in the Xiamen Meteorological Bulletin from 2005 to 2015 are calculated, and then GIS software is used to simulate the flood disaster risk under the multi-year average annual precipitation scenario (see Figure 7). Under this scenario, the areas with higher flood levels in Xiamen are mainly distributed in the northern mountainous areas. This is mainly due to the higher terrain in the north, the large terrain fluctuations, and the higher risk in the areas with higher precipitation. In the built-up area, the terrain is relatively flat, and various types of infrastructure are well-established. Therefore, the floods analysis level of this region is low. Overall, under this scenario, the overall flood disaster risk in Xiamen is relatively low.

• Flood risk assessment under the average precipitation scenario in flood season.

By taking the average precipitation of each site recorded in the Xiamen Meteorological Bulletin from 2005 to 2015 in the flood seasons (May to October of each year), we obtain the floods risk under the average precipitation situation in Xiamen for many years (see Figure 7). Under this scenario, the overall risk level of flood disaster in Xiamen has increased compared with scenario 1. The areas with high-risk level are still in the northern mountainous areas, but the medium-risk areas in the central and central urban areas have increased significantly. Overall, the flood risk level of the built-up area of Xiamen is still low, indicating that Xiamen can better cope with the flood threat caused by the regular disasters.

• Flood risk assessment under large typhoon precipitation scenarios.

According to the data in the Xiamen Statistical Meteorological Bulletin, during Typhoon Meranti in 2016, there was a strong precipitation in Xiamen. The cumulative precipitation in the whole process was 201.2 mm, which reached the magnitude of heavy storms. The typhoon caused serious casualties and direct economic losses. Typhoon Meranti is the most serious typhoon in Xiamen’s history, and it is used to simulate the flood disaster risk of Xiamen under extreme precipitation (see Figure 7). It is found that, unlike under Scenario 1 and Scenario 2, the overall flood risk level faced by Xiamen is significantly increased, which is the inevitable result of the increased risk of hazard factors. In addition, results show that the risk level of flood disasters is the highest in the central area, Haicang District, and Jimei District, which feature the highest economic development. On the one hand, it reflects the weakening of the original natural ecological adjustment capacity of the city due to the large-scale urbanization construction. On the other hand, it reflects that the existing infrastructure construction and fortification standards in Xiamen cannot cope with the flood disaster caused by extreme weather.

**Figure 8:**
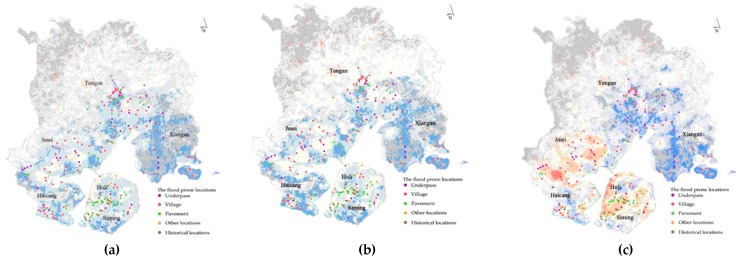
The flood prone locations under different scenarios in Xiamen: (**a**) the flood prone locations under the average annual precipitation scenario; (**b**) the flood prone locations under the average precipitation during the flood season scenario; and (**c**) the flood prone locations under the precipitation during the typhoon scenario.

By superimposing the flood risk results of the three precipitation scenarios with the flood disaster data in history (The existing disaster database records the floods in the urban built-up area of Xiamen City. Therefore, the recorded flood prone locations are also distributed in urban built-up areas. Meanwhile, in this paper, when simulating the flood prone locations in different scenarios, it is mainly targeted at urban built-up areas.), we further simulate the flood prone locations in the urban built-up area of Xiamen under different precipitation scenarios. As shown in Figure 8, the flood prone locations in Xiamen are mainly distributed within the urban built-up area, and the flood prone locations distributed in the central urban area are more likely to occur than other areas. Areas such as underpass interchanges, underground spaces and urban villages have become new distribution areas for flood disasters. In addition, comparing the flood prone locations under three precipitation scenarios, we can find that the flood disaster caused by large-scale typhoon precipitation is more likely to occur than the other two scenarios. This is due to the large-scale typhoon bringing short-term heavy precipitation, and the planning and construction standards for the drainage network in Xiamen are generally low. In particular, some old urban areas (e.g., the old towns and the urban villages in Tongan District and Jimei District) still use rainwater and sewage combined drainage system. Rainwater pipes are generally designed and constructed according to the labeling of one to two years, and the drainage capacity is seriously insufficient, leading to urban flood disaster.

### 4.6. Division of Flood Control Zones

According to the flood disaster risk assessment results obtained under different simulation scenarios and the flood disaster prone locations, flood control zones can be divided. When delineating the flood control zones, on the one hand, we take the urban construction land as the basis, and comprehensively consider the factors such as the layout of the water conservancy project, the flood prone locations and the administrative authority. On the other hand, we fully consider factors such as water system, terrain, and the drainage network. While maintaining the integrity of the water system, we rationally arrange the drainage methods in different terrain height areas to avoid dividing the high-lying and easily drained areas and low-lying areas into the same flood control zone. It should be noted that the flood control zones are also targeted at urban built areas. By dividing the flood control zones, a relatively independent and coordinated flood control system can be formed, which will provide a basis for us to formulate corresponding disaster prevention measures, coordinate the construction of flood control facilities between different districts, and to propose corresponding plans. In the end, it will help to form a comprehensive flood disaster prevention and control pattern with convenient management, intensive construction, strong resilience, and rapid response.

**Figure 9:**
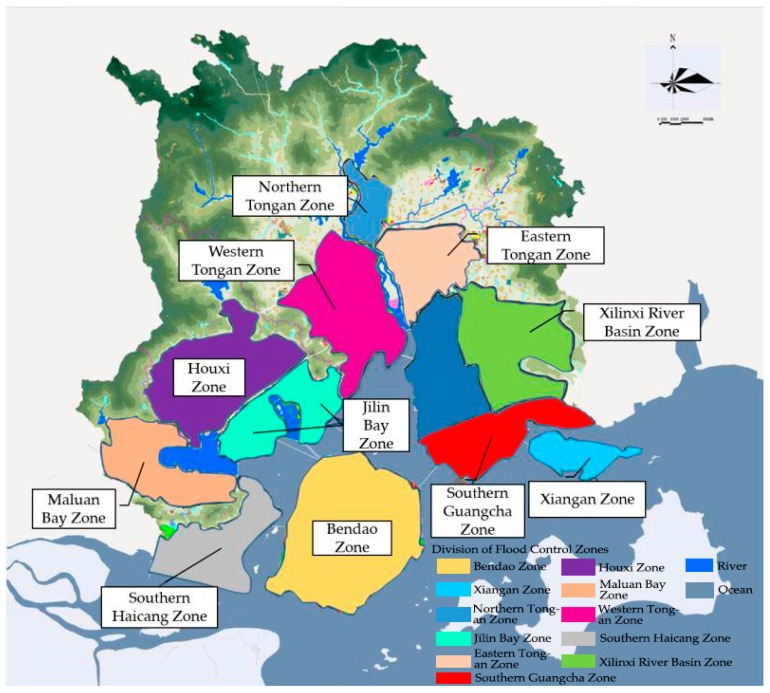
Division of flood control zones in Xiamen.

The terrain fluctuations in the north and south of Xiamen are large, the water system is complex, and the spatial and temporal distribution of precipitation is uneven, resulting in a large area affected by flood disasters in Xiamen. In this paper, we divide the built-up area of Xiamen into 11 flood control zones (see Figure 9), which includes the Bendao Zone, Northern Tongan Zone, Western Tongan Zone, Eastern Tongan Zone, Xilinxi River Basin Zone, Dongken Bay Zone, Southern Guangcha Zone, Xiangan Zone, Jilin Bay Zone, Houxi Zone, Maluan Bay Zone, and Southern Haicang Zone. On the basis of the division of flood control zones, each flood control zone can form a relatively independent flood control system by arranging flood control measures. In addition, within each flood control zone, we divide a number of drainage zones according to the water system and the terrain to form an efficient and intensive integrated drainage system.

## 5. Discussion

The FDRA facilitates the identification of the flooding risk environment in the city and the results of FDRA can also be used to guide safe urban development and construction. During urban planning and construction, the adaptability of cities to flood disasters should focus on two aspects [47]: (1) the spatial layout and development direction of the city should be adjusted to minimize the risk of flood disasters and enhance the city’s security; and (2) the disaster prevention system for urban floods should be based on the disaster risk assessment results and we should plan for various types of refuge facilities and shelters for different disaster risk levels to improve the city’s ability to respond to disasters. On this basis, on the one hand, we need to further improve the safety standards of urban flood control and drainage systems to reduce the risk level of flood disaster. On the other hand, we need to propose more effective disaster preventions and emergency response mechanisms to alleviate the loss of people’s property caused by disasters and provide a strong shield for the safe development of the city. In light of the “Safe Development” (The report of the 19th National Congress of the Communist Party of China, released on 18 October 2017, pointed out that it is necessary to establish a safe development concept and improve the public safety system. At the same time, it is necessary to curb serious accidents and improve the city’s ability to prevent and mitigate disasters) concept put forward by the Chinese central government, the “Beautiful Xiamen” development strategy proposed by the local government of Xiamen (on 8 September 2013, Xiamen City released the “Beautiful Xiamen Strategic Plan”. In the plan, it is proposed to rely on the natural pattern of the back mountain in Xiamen, improve the coordinated remediation of the basin, coordinate the key water supply engineering system in the basin, and build a regional ecological security pattern). Additionally, the relevant requirements in the overall urban planning of Xiamen, we put forward the relevant strategies for the flood disaster response in Xiamen (beginning in April 2018, Xiamen City’s overall urban planning revision work was initiated. The revision work has added research topics on urban disaster assessment and resilience enhancement, and made specific requirements for improving urban disaster response capabilities):

• Improve urban flood control standards and standards for prevention and control of internal hygiene

Based on the population density, economic development level, and existing engineering construction standards of various flood control zones, we believe that the Xiamen City flood control standard should be set to once in 50 years, and those areas that do not meet the standards need to be transformed and upgraded. In terms of rainwater pipe network standards, we believe that it should be designed according to the standards of one year and above. In addition, higher design standards should be adopted for areas with large catchment areas, important urban roads, and sinking areas.

• Improve the standards and requirements for lifeline engineering construction

The lifeline project includes four aspects of water supply lifeline engineering, power supply lifeline engineering, communication lifeline engineering, and rescue lifeline engineering. For the water supply lifeline project, we must ensure the safety of the water supply to the standby water source in the event of a flood disaster. At the same time, due to the internal sputum caused by seawater jacking in Xiamen, it is necessary to ensure the safety of drinking water quality in the event of flooding. For power lifeline projects, the level of flood risk at the selected location should be considered when deploying new power supply facilities. For the existing power supply facilities, it is necessary to raise the corresponding fortification standards to ensure the normal operation of the power system in the face of flood disaster. For the communication lifeline project, it is necessary to upgrade the standards for various communication facilities and improve the corresponding emergency plans. Ensure that the danger is notified in time prior to the disaster, and that the disaster can assist in disaster relief and rescue after the disaster. For the rescue lifeline project, it is necessary to ensure that the site selection safety and fortification standards of various medical and health service facilities meet the standards. For facilities in high-risk areas, it is necessary to re-evaluate their disaster prevention capabilities and carry out corresponding transformation and upgrading.

• Establish and improve the command system for flood control and drainage

First of all, it is necessary to construct a regional flood control and drainage command system in Xiamen to realize the automation and modernization of flood control dispatching in the city area, and to achieve unified dispatching and command of flood control dispatching work within the city area. Second, an organization in charge of food disasters needs to be established. Through the cooperation of different levels of government departments, we ensure the orderly development of disaster relief command work. Specifically, the municipal command department of local government should is responsible for the command of flood prevention and unify coordination of different departments to carry out rescue work. The district and county departments accept the unified leadership of the municipal departments, actively cooperate with the rescue work of the municipal departments, and reasonably allocate the duties and tasks of the towns (streets) within the district. All town (community) departments fully integrate grassroots comprehensive emergency resources and make overall arrangements for disaster prevention and relief efforts.

• Improve the flood response mechanism

All levels of departments should improve the corresponding flood emergency response plan, and pay attention to the effective docking and effect efficiency of the work between the different governments and departments. At the same time, through regular disaster response drills, the response mechanism of flood disaster can be constantly adjusted and improved in practice. In addition, we can rely on the disaster warning information platform to achieve real-time monitoring of flood disasters, and issue early warnings in the very beginning, and inform all departments and people to conduct evacuation work and disaster preparedness.

• Improve the construction of the materials storage

City-level disaster relief materials storage should be equipped with equipment such as fire, high-power pump, rubber boat, life jacket and other emergency materials. Each flood control zone should improve the materials according to the respective construction conditions, and it can be combined with emergency shelters and storage supermarkets. The disaster relief materials storage at all levels should raise the corresponding fortification standards to ensure the normal use of materials in the face of disasters.

• Strengthen the publicity and education of flood disaster knowledge

On the one hand, it is possible to carry out more comprehensive flood prevention and disaster prevention knowledge publicity through various levels of government organizations. In particular, it is possible to strengthen the popular science work for vulnerable groups, such as the elderly and children, through community activities centers and school education. On the other hand, we should actively build a mobile phone information platform and make full use of the advantages of the information age to carry out disaster prevention publicity and education.

## 6. Conclusions

China is one of the countries frequently affected by flood disasters, especially in coastal areas. The flooding occurrences have caused tremendous loss of life and damage to property in urban areas, thwarting economic and social development of cities. Urban flood disaster risk assessment is the basis for urban flood control and drainage-related work and disaster prevention and mitigation schemes. It is of great theoretical and practical significance to explore flood disaster risk assessment methods in the context of frequent flood disasters. Through flood disaster risk assessment, we can effectively identify the degree of risk in different areas of the city, thus laying the foundation for the formulation of urban disaster prevention planning and the response strategy.

In this paper, we refer to the existing indicators of flood disaster assessment, and construct the index system of urban flood risk assessment from the four perspectives: hazard of disaster factors, sensitivity to environment, vulnerability of the disaster-bearing body and the city’s own disaster prevention capability and resilience. Then we add the precipitation factor as a scenario parameter to the assessment of flood disaster, in order to assess the flood disaster risk under annual average precipitation scenarios, multi-year flood season average precipitation scenarios and large typhoon precipitation scenarios. Taking Xiamen as an example, we assessed the flood disaster risk of Xiamen under different precipitation scenarios, and simulated the occurrence of flood disaster in Xiamen under different scenarios. Based on the results of the simulation, we further proposed the corresponding coping strategies.

Our studies expand the research on flood disaster risk assessment, and also provide reference for relevant cities to deal with flood disaster. Although the method proposed in this paper realizes the urban flood disaster risk assessment under different scenarios it is, however, still a static assessment, and is impossible to realize real-time monitoring and dynamic assessment of urban flood disasters. In the following research, we will further deepen the methods of simulating flood disaster scenarios and methods of flood disaster risk assessment, and continuously expand the research methods and scale of flood disaster risk assessment. Simultaneously, through more detailed data collection and acquisition, we will explore the risk assessment of flood disasters at smaller scales.

## Figures and Tables

**Table 1:** Index system for evaluating the flood disaster risk of Xiamen.

Category	Sub-Category	Explanation ^1,2^	Attribute	Weight
Hazard of disaster factors	Precipitation factor	Annual average precipitation, mean precipitation during flood season and precipitation during typhoon (unit: mm)	Positive	0.1786
	Flood factor	Distance to the river (unit: m) and reservoir storage capacity (unit: m^3^)	Positive	0.0815
Sensitivity to the environment	Terrain factor	Elevation (unit: m) and slope (unit: degree)	Positive	0.1538
	Land factor	The proportion of cultivated land (unit: %)	Negative	0.0393
	River network factor	River network density (unit: %)	Positive	0.0949
Vulnerability of the disaster-bearing body	Population vulnerability	Population density (unit: %)	Positive	0.0387
	Economic vulnerability	Per capita GDP (unit: CNY)	Positive	0.0274
	Building vulnerability	Building quality (unit: null) and architectural age (unit: year)	Positive	0.0531
Disaster prevention and resilience	Flood control capacity	Rainwater pipe network density (unit: %)	Negative	0.1762
	Comprehensive response ability	Urbanization level (unit: %) and road network density (unit: %)	Negative	0.1564

^1^ Data sources: The economic, population, and other data involved in this paper are from Xiamen Statistical Yearbook and Xiamen City Economic Census Data. Data on precipitation, floods, and typhoons are collected from the Xiamen Meteorological Bureau, the Oceanic Administration, and the Emergency Office. The relevant data on land and topography are from the Xiamen Municipal Bureau of Land and Resources and the Earthquake Administration. The relevant data of the road network and other municipalities come from the Xiamen Municipal Transportation Bureau, the Housing Construction Bureau, and the Statistical Yearbook. ^2^ Note: In parentheses are the units of each indicator.
